# Online Dietary Intake Estimation: The Food4Me Food Frequency Questionnaire

**DOI:** 10.2196/jmir.3105

**Published:** 2014-06-09

**Authors:** Hannah Forster, Rosalind Fallaize, Caroline Gallagher, Clare B O’Donovan, Clara Woolhead, Marianne C Walsh, Anna L Macready, Julie A Lovegrove, John C Mathers, Michael J Gibney, Lorraine Brennan, Eileen R Gibney

**Affiliations:** ^1^UCD Institute of Food and HealthUniversity College DublinDublinIreland; ^2^Hugh Sinclair Unit of Human Nutrition and Institute for Cardiovascular and Metabolic ResearchUniversity of ReadingReadingUnited Kingdom; ^3^Human Nutrition Research CentreInstitute for Ageing and HealthNewcastle UniversityNewcastleUnited Kingdom

**Keywords:** food frequency questionnaire, online dietary assessment tool, Food4Me, dietary assessment, Web-based

## Abstract

**Background:**

Dietary assessment methods are important tools for nutrition research. Online dietary assessment tools have the potential to become invaluable methods of assessing dietary intake because, compared with traditional methods, they have many advantages including the automatic storage of input data and the immediate generation of nutritional outputs.

**Objective:**

The aim of this study was to develop an online food frequency questionnaire (FFQ) for dietary data collection in the “Food4Me” study and to compare this with the validated European Prospective Investigation of Cancer (EPIC) Norfolk printed FFQ.

**Methods:**

The Food4Me FFQ used in this analysis was developed to consist of 157 food items. Standardized color photographs were incorporated in the development of the Food4Me FFQ to facilitate accurate quantification of the portion size of each food item. Participants were recruited in two centers (Dublin, Ireland and Reading, United Kingdom) and each received the online Food4Me FFQ and the printed EPIC-Norfolk FFQ in random order. Participants completed the Food4Me FFQ online and, for most food items, participants were requested to choose their usual serving size among seven possibilities from a range of portion size pictures. The level of agreement between the two methods was evaluated for both nutrient and food group intakes using the Bland and Altman method and classification into quartiles of daily intake. Correlations were calculated for nutrient and food group intakes.

**Results:**

A total of 113 participants were recruited with a mean age of 30 (SD 10) years (40.7% male, 46/113; 59.3%, 67/113 female). Cross-classification into exact plus adjacent quartiles ranged from 77% to 97% at the nutrient level and 77% to 99% at the food group level. Agreement at the nutrient level was highest for alcohol (97%) and lowest for percent energy from polyunsaturated fatty acids (77%). Crude unadjusted correlations for nutrients ranged between .43 and .86. Agreement at the food group level was highest for “other fruits” (eg, apples, pears, oranges) and lowest for “cakes, pastries, and buns”. For food groups, correlations ranged between .41 and .90.

**Conclusions:**

The results demonstrate that the online Food4Me FFQ has good agreement with the validated printed EPIC-Norfolk FFQ for assessing both nutrient and food group intakes, rendering it a useful tool for ranking individuals based on nutrient and food group intakes.

## Introduction

Associations between dietary behaviors and chronic health risks have been established on numerous occasions [1,2], making the ability to measure dietary intake crucial for researchers and public health practitioners to improve health outcomes [3,4]. Dietary assessment methods are used for quantification of both short- and long-term (habitual) dietary intakes, and are essential tools in epidemiological investigations and intervention studies assessing relationships between diet and health in both population and clinical settings [3,5].

Food records (or diaries), 24-hour recalls, and food frequency questionnaires (FFQ) are the three principle assessment methods that are used traditionally to measure dietary intake [6,7]. Food records require respondents to record all foods and beverages consumed over a specified period of time, generally between 3 and 7 days prospectively [8-10]. The 24-hour recalls are interviewer led and entail asking the respondent to remember and record all foods consumed in the preceding 24-hour period [7,10,11]. FFQs are also retrospective assessment tools and require respondents to report the frequency of consumption of a predefined list of foods over a prolonged period of time, typically the previous 6 or 12 months [7].

Internet availability and usage has increased globally over the past decade and, as a result, traditional methods of dietary assessment have been modified for online and electronic use in both research and industry [12]. Compared with traditional methods of dietary assessment, online methods allow for the automatic storage of input data and the automatic generation of nutritional outputs [13]. While traditional methods of dietary assessment can be supplemented with food photograph atlases to aid portion size recognition and estimation [14], online methods of dietary assessment can be designed to incorporate food photographs, making them more convenient for users to complete [15-17]. Furthermore, compared with traditional methods, online dietary assessment methods can be used to target specific geographical population groups, can be accessed remotely, and can be designed to be easy to complete.

The strengths and weaknesses of traditional dietary assessment methods are well documented [8,18-21]. The quality of data from any dietary assessment method, traditional or online, will depend ultimately on the respondent’s accuracy in recording the required details [22]. As a result, online food diaries may have greater use commercially or for small dietary studies as they require respondents to be highly motivated to record data [22].

FFQs and 24-hour recalls are the most commonly used approaches for assessing dietary intake for large population studies [22]. Although Web-based 24-hour recalls have been demonstrated to show good agreement with traditional methods on numerous occasions [23-25,26], they are largely limited by the day-to-day variability in dietary intake and may not accurately assess intakes of foods that are eaten infrequently (eg, oily fish). As a result, multiple 24-hour recalls over several non-consecutive days are required to reflect usual dietary intake [22]. Unlike food records and 24-hour recalls, FFQs can capture long-term dietary intake in a single administration and are less cumbersome to complete [27-29]. Although FFQs have often been reported to bear the greatest amount of measurement error, not only when considering under-reporting but also over-estimation of dietary intakes [30], they have been shown to have good validity for ranking nutrient intakes on numerous occasions [20-22,31]. As a result, they can be used to categorize nutrient intakes as “low”, “recommended”, or “high” compared with recommended intakes, rendering them invaluable tools for assessing nutritional intake status [20-21].

In recent years, many well-established FFQs have been developed into Web-based versions and there is a growing body of evidence demonstrating that data from Web-based FFQs are comparable with data from printed versions and/or have good validity with reference methods such as 24-hour recalls and food diaries [15-17,32]. Web-based FFQs possess many benefits over printed questionnaires: they are more cost-effective, they can be pre-programmed to ensure all questions are answered, and photographs can be incorporated to enhance food recognition and portion size estimation [15,17,32].

The present study was conducted as part of the EU 7th European Framework Programme “Food4Me” project [33]. The Food4Me project aims to investigate the potential of, and public attitudes toward, personalized nutrition and is the first study of its kind designed to emulate an entirely Web-based, personalized nutrition service [34].

The objectives of the present study were to develop an online FFQ for dietary data collection in the Food4Me study and to compare estimates of intakes obtained using this tool with those obtained from the validated European Prospective Investigation of Cancer (EPIC) Norfolk printed FFQ [35].

## Methods

### Development of the Online Food4Me Food Frequency Questionnaire

#### Overview

The online Food4Me FFQ was designed to assess food and nutritional intake across seven centers in Europe, as part of a dietary intervention study within the Food4Me project [33]. The design and development of the novel online Food4Me FFQ was led by researchers at University College Dublin and software company Creme Global (Dublin, Ireland).

The well-validated EPIC-Norfolk FFQ (version CAMB/PQ/6/1205) [35] was used as a guide for food items and food group categories. In developing the Food4Me FFQ, the original 130 food items presented in the EPIC-Norfolk FFQ were expanded upon to incorporate an additional 27 commonly consumed food items that were considered nutritionally important across the seven EU countries in the Food4me study. In expanding the food list, some food items were added (eg, tortillas, wraps) to existing food categories; some new foods were added to existing food items (eg, “noodles and cous cous” were added to “white pasta or green pasta”), and, in some cases, existing food items were split into more defined types (eg, “oily fish, fresh or canned” was split into “non-smoked oily fish, canned” and “non-smoked oily fish, fresh”). Standardized color photographs were incorporated into the online Food4Me FFQ to facilitate accurate portion size estimation. As in the printed EPIC-Norfolk FFQ, the 157 food items were divided into 11 categories viz, “cereal”, “bread and savory biscuits”, “potatoes, rice, and pasta”, “meat and fish”, “dairy products”, “fats and spreads”, “sweets and snacks”, “soups, sauces, and spreads”, “drinks”, “fruit”, and last, “vegetables”. In addition, the Food4Me FFQ included an additional section on dietary habits with further questions relating to additional foods consumed, the addition of salt to foods, consumption of fried foods, and supplement use (as in the EPIC-Norfolk FFQ).

**Figure 1:**
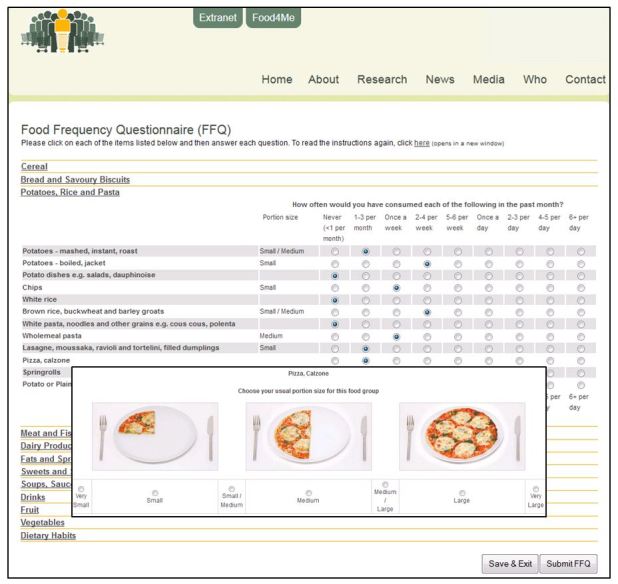
Screenshot of the online Food4Me Food Frequency Questionnaire.

Frequency of intake was estimated by asking, “How often would you have consumed each of the following in the past month?” and participants could select their frequency from nine categories of intake ranging from “never (<1 per month)” to “6+ per day”. After selecting their frequency of consumption, participants were asked to choose their usual serving size from a range of portion size pictures for each food item (see Figure 1). The online Food4Me FFQ was pre-programmed to ensure that a frequency of consumption was reported for every food item before the participant could submit the FFQ and was designed so that participants could check and/or modify previous responses before submitting the FFQ. Illustrations of the Food4Me FFQ are shown in Figure 1 and Multimedia Appendix 1.

#### Nutritional Composition and Portion Sizes

Nutritional composition and portion sizes were calculated from the 2008-2010 National Adult Nutrition Survey (NANS) database, which consists of detailed dietary intake data for 1500 Irish adults [36].

The nutritional composition of the 157 food items, listed in the Food4Me FFQ, was derived from the wider list of corresponding foods within the NANS database. From these, the most frequently consumed foods were identified and used to calculate the composition of the list of foods in the Food4Me FFQ. For example, for all pizzas consumed in the NANS dataset, the nutritional composition per 100g was computed for the three most frequently consumed and the mean of these was then calculated to give a single nutritional composition for pizza. The nutritional composition for the NANS dataset was analyzed using WISP (Tinuviel Software, Anglesey, UK). WISP uses the 5^th^and 6^th^editions of McCance and Widdowson’s *The Composition of Foods*, plus all nine supplemental volumes to generate nutrient intake data [37,38]. In addition, the nutritional composition dataset was modified previously and updated to include recipes of composite dishes, generic commercial foods, new foods on the market, and current manufacturers’ information. For additional foods unique to a specific European country in the Food4Me study, such as Greek baklava, the relevant national food composition database was used.

For the calculation of portion sizes, the food codes for each of the frequently consumed foods identified from the NANS database were merged and recoded into a single food code for each food item listed in the FFQ. Using the newly assigned food code, PASW Statistics version 18 (SPSS Inc Chicago, IL, USA) was used to calculate the 25^th^, 50^th^, and 75^th^percentiles of daily intake, which represent small, medium, and large portion sizes of these foods when consumed in the free living population. Options for portion sizes above, below, and in between these percentiles were also provided to accommodate the wide variability in portion size across populations.

#### Photographs

Foods were purchased from local supermarkets and bakeries in Dublin. All foods were prepared and photographs taken in the Institute of Food and Health, University College Dublin. Food photographs were taken by a professional photographer over 5 days/sessions. A standard dining set of plates and cutlery, positioned uniformly with the same lighting, was used for each session. The calculated NANS portion sizes were used as a guideline and all foods were weighed out using calibrated portable food scales (Tanita, Japan).

### Study Sample and Study Design

#### Overview

The comparison of the online Food4Me FFQ with the printed EPIC-Norfolk FFQ was conducted in two centers involved in the Food4Me study (University College Dublin and University of Reading) using the English language version of the questionnaire (consisting of 157 food items). The study received approval from the ethical committees at both universities and was carried out between March and October 2012 (LS-11-118-Gibney-Walsh and 01/12-Lovegrove respectively). Participants (n=177) aged ≥18yrs were recruited at both centers. Participants were provided with an information sheet explaining the study, signed a consent form, and completed a brief screening questionnaire either in person or by post. Weight and height were self-reported. Individuals with self-reported/diagnosed food intolerances/allergies or those receiving dietary advice were ineligible to participate.

Eligible participants completed both the online Food4Me FFQ and the printed EPIC-Norfolk FFQ in random order. The printed EPIC-Norfolk FFQ was modified to ask participants’ about their food intake over the past month rather than over the past year. To minimize possible effects of temporal changes in dietary intake, participants with more than 4 weeks between completing both FFQs were excluded from analyses. The printed EPIC-Norfolk FFQ was delivered to the participant in person or by post. The Food4Me FFQ was accessed via a hyperlink to the website sent in an email containing the participant’s individual username and password.

#### Under-Reporters

The Henry equation was used to calculate basal metabolic rate (BMR) and BMR was multiplied by 1.1 to calculate the lowest possible estimated energy requirements (EER) for each participant [39]. Participants reporting energy intakes lower than their EER were classified as under-reporters.

### Dietary Intake Analysis

Printed EPIC-Norfolk questionnaires were coded using the specified template format and cross-checked before sending to Strangeways Research Laboratory (University of Cambridge) for processing using FETA software [40]. The nutritional composition database used in the EPIC-Norfolk FFQ is based on the revised and extended 5^th^edition of McCance and Widdowson’s *The Composition of Foods* plus supplemental volumes [41]. The Food4Me FFQ nutritional intake data was generated automatically by the online Food4Me programmed system, as described above. For the purpose of the current study, consumption of dietary supplements was not included in the analyses.

Data were imported into SPSS for analysis and descriptive statistics were computed to describe the general characteristics of participants. Mean nutrient intakes and standard deviations were determined for both FFQs. General linear model analysis controlling for energy was used to compare nutrient intakes between the FFQs. Correlation coefficients were computed to assess the association between the two methods. The relative agreement between the two FFQs was assessed using cross-classification of nutrient intakes to estimate the percentage of participants who were classified by the two methods into quartiles of “exact agreement”, “exact agreement plus adjacent”, “disagreement”, and “extreme disagreement”. Bland and Altman analysis was performed for the macronutrients to assess the limits of agreement between the two FFQs. For each macronutrient, the differences of the mean between the two methods (EPIC−Food4Me) were plotted against the average of the two methods ([EPIC+Food4Me]/2). Methods were considered comparable if greater than 95% of data plots lay within the limits of agreement (mean ± 2SD).

Differences in food group intakes between the two methods were examined. To do this, the food items in the EPIC-Norfolk and Food4Me FFQs were arranged into 35 food groups. Independent samples *t* tests were used to compare daily food group intakes between the two FFQs. Bland and Altman analysis was performed for the food groups to assess the limits of agreement between the two FFQs. Spearman’s correlation coefficients (SCC) were computed to assess the associations between the two methods for the daily intake of each of the 35 food groups. To assess the relative agreement between the two methods for daily food group intake, food group intakes were cross-classified to estimate the percentage of participants classified by the two methods into quartiles of “exact agreement”, “exact plus adjacent agreement”, and “disagreement”. All data were analyzed using PASW Statistics version 18 (SPSS Inc Chicago, IL, USA). *P*<.05 was considered statistically significant. GraphPad PRISM version 6 was used to produce the Bland and Altman plots (GraphPad Software, Inc California, USA).

## Results

### Overview of the Study Population

**Figure 2:**
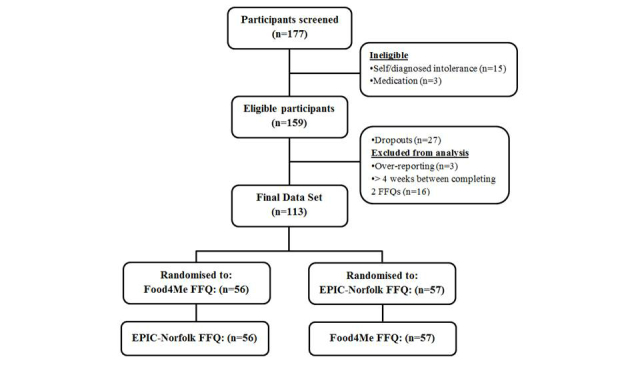
Flow of participants through the study. FFQ: food frequency questionnaire.

A total of 177 participants were screened to participate in the study with 159 eligible for inclusion. Following initiation of the study, 27 participants dropped out, as shown in Figure 2. Reasons for dropouts included external commitments, for example, holidays and technical issues. A further 19 participants were excluded from the analysis: 16 had >4 weeks between completion of the two FFQs and 3 reported energy intakes >4500 kcal per day with the Food4Me FFQ (considered to be unrealistically high) [42]. The final data set, therefore, consisted of 113 participants as illustrated in Figure 2. The results presented here are for the dietary assessment of 67 females (59.3%) and 46 males (40.7%) who completed both the printed EPIC-Norfolk FFQ and online Food4Me FFQ in random order.

Demographic characteristics of the study population are presented in Table 1. Overall, for all participants, there were no significant differences in age for males and females; however, self-reported body mass index (BMI) was significantly lower for females compared with males. As shown in Table 1, significant differences in age, weight, and BMI were observed across the two centers. The participants recruited in Dublin were significantly older (*P*<.005), were significantly heavier (*P*<.005), and had higher BMI (*P*<.05) than those recruited in Reading.

**Table 1:** Demographic characteristics of the study population in total and across centers, by gender.

Participants			Demographic characteristics, mean (SD)		
		n	Age (y)	Weight (kg)	BMI (kg/m^2^)^a^
**All Participants**					
	Male	46	32.0 (12.6)	77.3 (11.3)	24.3 (3.0)
	Female	67	29.0 (8.0)	62.2 (9.4)	22.6 (2.6)^d^
	All	113	30.0 (10.2)	68.4 (12.6)	23.3 (2.9)
**Dublin Participants**					
	Male	32	35.1 (13.0)	78.6 (10.6)	24.8 (2.8)
	Female	32	30.2 (7.2)	64.3 (8.9)	23.1 (2.5)^e^
	All	64	32.6 (10.7)	71.5 (12.1)	24.0 (2.8)
**Reading Participants**					
	Male	14	24.2 (7.6)^b^	74.3 (12.4)	23.1 (3.2)
	Female	35	27.9 (8.6)	60.3 (9.4)	22.2 (2.6)
	All	49	26.9 (8.4)^b^	64.3 (12.1)^b^	22.5 (2.8)^c^

^a^Body mass index (BMI) based on self-reported weight and height.

^b^Significantly different between centers, *P*<.005.

^c^Significantly different between centers, *P*<.05.

^d^Significantly different from males, *P*<.005.

^e^Significantly different from males, *P*<.05.

### Comparison of Nutrient Intakes Between the Two Questionnaires

Mean energy and nutrient intakes estimated from the two FFQs are presented in Table 2. Energy intakes were significantly higher for the Food4Me FFQ in comparison with the EPIC-Norfolk FFQ (*P*<.001). However, when the occurrence of under-reporting was examined, 58 participants were classified as under-reporters with the EPIC-Norfolk FFQ compared with only 20 participants for the Food4Me FFQ. Of the 20 participants’ under-reporting in the Food4me FFQ, 18 also under-reported using the EPIC-Norfolk FFQ (90%).

After controlling for energy, intakes of macronutrients showed relatively good agreement with no significant differences in energy derived from total fat, saturated fatty acids (SFA), monounsaturated fatty acids (MUFA), or carbohydrates. Significant differences were observed in both total polyunsaturated fatty acid intake and % energy derived from polyunsaturated fatty acids (PUFA), at *P*<.001. Examination of differences at a micronutrient level demonstrated no significant differences between the two FFQs for intakes of calcium, vitamin B12, and vitamin D. Significant differences were observed for intakes of folate, iron, carotene, riboflavin, thiamin, vitamin B6, vitamin C, vitamin A (RE), retinol, vitamin E, and sodium (Table 2). However, after controlling for energy and, where appropriate, center, randomization group and/or gender, vitamin C and vitamin A (RE) were no longer significantly different (Table 2). The removal of under-reporters reduced the agreement between the two FFQs for total fat (% total energy [TE]), but improved the agreement for protein (% TE) showing no significant difference when controlling for energy, center, and gender (both alone and in combination), as shown in Table 3.

**Figure 3:**
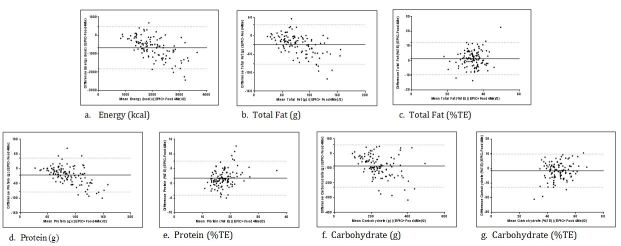
Bland and Altman plots with mean difference and limits of agreement (solid line represents mean difference and dotted lines represent limits of agreement). TE: total energy.

Bland and Altman plots for mean daily energy, total fat, protein, and carbohydrate intakes are presented in Figure 3. The Bland and Altman plot for energy indicated broad limits of agreement. The mean difference in estimated energy intake between FFQs was relatively high (676 kcal/d) with greater energy intakes recorded using the Food4Me FFQ compared with the EPIC-Norfolk FFQ. In addition, the difference in estimates of energy intakes between FFQs became progressively greater with higher mean intakes (Figure 3). Despite this, less than 5% of cases fell outside the limits of agreement for energy (n=5), confirming an acceptable level of agreement between the two methods.

As illustrated in Figure 3, similar to energy, the difference in estimates of total fat (g) and protein (g) intakes between the two FFQs became progressively greater with higher mean intakes. Poor levels of agreement between the methods were observed for the macronutrients, with greater than 5% of cases falling outside the limits of agreement for protein (g) (n=8), carbohydrate (g) (n=6), and total fat (g) (n=6). After adjusting for energy, the agreement between the two methods improved, as less than 5% of cases fell outside the limits of agreement for both carbohydrate (% TE) (n=4) and total fat (% TE) (n=4) (Figure 3). Although energy adjustment improved the distribution of cases for protein, more than 5% of cases (n=7) remained outside the limits of agreement [43], indicating a poorer level of agreement between the methods for protein (% TE). Correlation coefficients for estimates of nutrient intakes between the two FFQs are presented in Table 4. The mean correlation coefficient for nutrient intake between the two FFQs was .60. Correlations varied from .43 (polyunsaturated fatty acids % TE) to .86 (alcohol). For micronutrients, correlation coefficients were lowest for thiamin (.46) and highest for vitamin C (.69). The cross-classification of mean daily intakes between the two FFQs are also shown in Table 4. The percentage of participants classified into quartiles of “exact agreement” varied from 37% (polyunsaturated fatty acids %TE) to 63% (alcohol). The percentage of participants cross-classified into quartiles of “exact agreement plus adjacent” was lowest for polyunsaturated fatty acids (77%) and highest for alcohol (97%). Although the mean percentage of participants classified into quartiles of “disagreement” (opposite plus extreme quartiles) was 15%, the mean percentage categorized into quartiles of “extreme disagreement” was 2.5%.

**Table 2:** Mean daily nutrient intakes estimated by printed EPIC-Norfolk FFQ^a^and online Food4Me FFQ (n=113).

Nutrient	EPIC-Norfolk FFQ,mean (SD)	Food4Me FFQ,mean (SD)	*P* value^b^	*P* value^c^
Energy (kcal)	1684.62 (483.41)	2356.08 (809.36)	-	-
Total Fat (g)	66.10 (24.27)	89.48 (38.23)	.001	.001
Total Fat (% TE^d^)	34.88 (6.18)	33.50 (5.31)	.07	.03
SFA^e^ (g)	24.91 (10.50)	36.03 (16.52)	.29	.29
SFA (% TE)	13.13 (3.40)	13.39 (2.78)	.52	.73
MUFA^f^(g)	23.26 (8.75)	32.82 (14.84)	.09	.09
MUFA (% TE)	12.26 (2.35)	12.27 (2.54)	.99	.99
PUFA^g^(g)	12.18 (6.33)	14.33 (5.97)	<.001	<.001
PUFA (% TE)	6.44 (2.63)	5.46 (1.15)	<.001	<.001
Protein (g)	75.53 (21.82)	96.92 (36.87)	.26	.91
Protein (% TE)	18.33 (4.24)	16.56 (3.35)	.001	.001
Carbohydrate (g)	197.09 (69.81)	281.89 (96.48)	.09	.12
Carbohydrate(% TE)	46.82 (8.14)	48.41 (6.91)	.11	.06
Total sugars (g)	103.92 (45.78)	130.00 (48.61)	.04	.001
Alcohol (g)	7.21 (9.10)	13.61 (15.72)	.18	.18
Calcium (mg)	835.03 (255.85)	1159.12 (459.22)	.17	.17
Total folate (µg)	250.16 (68.70)	375.16 (136.40)	<.001	<.001
Iron (mg)	9.70 (2.74)	15.91 (5.97)	<.001	<.001
Total carotene (µg)	3512.61 (2004.14)	5811.99 (4180.17)	.001	.02
Riboflavin (mg)	1.74 (0.47)	2.42 (0.92)	.02	.02
Thiamin (mg)	1.34 (0.37)	2.42 (1.66)	<.001	<.001
Vitamin B6 (mg)	2.03 (0.52)	2.84 (1.08)	.03	.01
Vitamin B12 (µg)	5.93 (2.79)	7.22 (3.24)	.49	.49
Vitamin C (mg)	107.12 (55.87)	164.50 (87.85)	.003	.11
Vitamin A (RE) (µg)	984.94 (470.95)	1735.23 (3712.62)	.04	.95
Retinol (µg)	385.73 (300.15)	445.73 (277.59)	.02	.02
Vitamin D (µg)	2.96 (1.90)	3.67 (2.15)	.44	.48
Vitamin E (mg)	10.64 (4.63)	10.71 (4.32)	<.001	<.001
Sodium (mg)	2442.27 (772.86)	2597.38 (1070.53)	<.001	<.001
Salt (g)	6.11 (1.93)	6.49 (2.68)	<.001	<.001

^a^FFQ: food frequency questionnaire.

^b^All *P* values were derived by controlling for energy using general linear model analysis.

^c^Controlled for energy and, where appropriate, center, gender, and randomization group using general linear model analysis. No significant interactions were observed between method and gender, center and randomization group.

^d^TE: total energy.

^e^SFA: saturated fatty acids.

^f^MUFA: monounsaturated fatty acids.

^g^PUFA: polyunsaturated fatty acids.

**Table 3:** Mean daily nutrient intakes estimated by printed EPIC FFQ^a^(n=55) and online Food4Me FFQ (n=93) with under-reporters removed.

Nutrient	EPIC (n=55),mean (SD)	Food4Me (n=93),mean (SD)	*P* value^b^	*P* value^c^
Energy (kcal)	2023.79 (402.71)	2573.63 (714.10)	-	-
Total Fat (g)	80.68 (20.15)	98.82 (35.18)	.01	.01
Total Fat (% TE ^d^)	35.84 (4.72)	34.11 (5.08)	.04	.04
SFA^e^(g)	30.43 (9.44)	39.88 (15.46)	.60	.60
SFA (% TE)	13.54 (3.02)	13.68 (2.71)	.76	.76
MUFA^f^(g)	28.51 (7.58)	36.33 (13.82)	.30	.30
MUFA (% TE)	12.65 (1.97)	12.55 (2.47)	.78	.78
PUFA^g^(g)	14.80 (5.29)	15.72 (5.57)	<.001	<.001
PUFA (% TE)	6.57 (1.97)	5.50 (1.13)	<.001	<.001
Protein (g)	85.69 (17.42)	105.73 (33.99)	.85	.30
Protein (% TE)	17.21 (3.29)	16.53 (3.26)	.22	.22
Carbohydrate (g)	239.40 (70.54)	306.18 (87.44)	.48	.48
Carbohydrate (% TE)	47.04 (7.61)	47.97 (6.94)	.45	.36
Total sugars (g)	131.41 (47.46)	141.51 (44.27)	.009	.001
Alcohol (g)	8.50 (11.60)	14.80 (16.84)	.33	.33
Calcium (mg)	956.48 (226.18)	1256.82 (442.11)	.14	.14
Total folate (µg)	285.67 (63.38)	403.13 (125.66)	<.001	<.001
Iron (mg)	10.91 (2.58)	17.18 (5.61)	<.001	<.001
Total carotene (µg)	4078.51 (2051.75)	6280.68 (4311.00)	.004	.004
Riboflavin (mg)	1.97 (0.41)	2.60 (0.90)	.03	.03
Thiamin (mg)	1.55 (0.35)	2.48 (1.48)	.001	.001
Vitamin B6 (mg)	2.32 (0.47)	3.09 (1.01)	.02	.01
Vitamin B12 (µg)	6.88 (2.93)	7.82 (3.20)	.72	.72
Vitamin C (mg)	126.64 (57.42)	178.47 (87.62)	.01	.11
Vitamin A (RE) (µg)	1190.08 (471.67)	1917.85 (4067.44)	.53	.53
Retinol (µg)	490.82 (323.56)	481.24 (287.30)	.01	.01
Vitamin D (µg)	3.48 (2.03)	3.98 (2.21)	.58	.58
Vitamin E (mg)	13.06 (4.56)	11.65 (4.07)	<.001	<.001
Sodium (mg)	2879.23 (712.59)	2841.48 (1009.77)	<.001	<.001
Salt (g)	7.20 (1.78)	7.10 (2.52)	<.001	<.001

^a^FFQ: food frequency questionnaire.

^b^All *P* values were derived by controlling for energy using general linear model analysis.

^c^Controlled for energy and, where appropriate, center and/or gender using general linear model analysis. No significant interactions were observed between method and gender or center.

^d^TE: total energy.

^e^SFA: saturated fatty acids.

^f^MUFA: monounsaturated fatty acids.

^g^PUFA: polyunsaturated fatty acids.

**Table 4:** Unadjusted correlation coefficients and cross-classification of quartiles of mean energy and nutrient intakes derived from the online Food4Me FFQ^a^and printed EPIC-Norfolk FFQ.

	Correlation^b^	Exact agreement^d^, (%)	Exact agreement + adjacent^e^, (%)	Disagreement^f^, (%)	Extreme disagreement^g^, (%)
Energy (kcal)	.68	52	88	12	1
Total Fat (g)	.70	46	90	10	1
Total Fat (% TE^h^)	.54^c^	39	78	22	4
SFA^i^ (g)	.71	46	91	9	1
SFA (% TE)	.63^c^	38	84	16	2
MUFA^j^(g)	.70	47	93	7	2
MUFA (% TE)	.57^c^	39	81	19	2
PUFA^k^(g)	.56	39	86	14	4
PUFA (% TE)	.43	37	77	23	1
Protein (g)	.63	46	86	14	1
Protein (% TE)	.63	50	85	15	2
Carbohydrate (g)	.63	55	84	16	3
Carbohydrate (% TE)	.72^c^	53	88	12	0
Total sugars (g)	.74	57	94	6	2
Alcohol (g)	.86	63	97	3	0
Calcium (mg)	.51	45	81	19	3
Total folate (µg)	.53^c^	49	81	19	3
Iron (mg)	.48	44	79	21	5
Total carotene (µg)	.58	44	80	20	2
Riboflavin (mg)	.52	38	82	18	4
Thiamin (mg)	.46	40	84	16	5
Vitamin B6 (mg)	.56	49	85	15	4
Vitamin B12 (µg)	.49	39	81	19	4
Vitamin C (mg)	.69	52	89	11	2
Vitamin A (RE) (µg)	.55	42	82	18	3
Retinol (µg)	.65	48	91	9	3
Vitamin D (µg)	.57	42	87	13	4
Vitamin E (mg)	.57	43	81	19	4
Sodium (mg)	.58	50	84	16	1

^a^FFQ: food frequency questionnaire.

^b^Correlation is significant at the .01 level (2-tailed) for all nutrients analyzed.

^c^Pearson’s correlation.

^d^Exact agreement: % of cases cross-classified into the same quartile.

^e^Exact + adjacent agreement: % of cases cross-classified into the same or adjacent quartile.

^f^Disagreement: % of cases cross-classified 2 quartiles apart.

^g^Extreme quartiles: % of cases cross-classified into extreme quartiles.

^h^TE: total energy.

^i^SFA: saturated fatty acids.

^j^MUFA: monounsaturated fatty acids.

^k^PUFA: polyunsaturated fatty acids.

### Comparison of Food Group Intakes Between the Two Questionnaires

To examine differences in food group intakes between the two FFQs, the food items in the EPIC-Norfolk FFQ and Food4Me FFQ were aggregated into 35 food groups. Mean daily intakes estimated from the two FFQs for the 35 food groups are presented in Table 5. Mean daily intakes for 17 of the 35 food groups analyzed were significantly higher for the online Food4Me FFQ in comparison with the printed EPIC-Norfolk FFQ.

**Figure 4:**
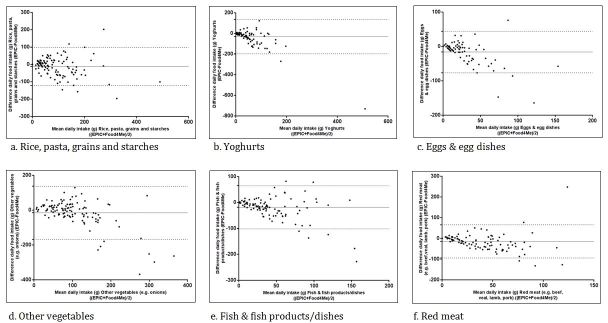
Bland and Altman plots for selected food groups with mean difference and limits of agreement (solid line represents mean difference and dotted lines represent the limits of agreement).

Bland and Altman analysis for daily food group intake was performed to examine the agreement between both methods. Overall, acceptable levels of agreement were observed for 15 of the 35 food groups, with less than 5% of cases (n<6) falling outside the limits of agreement. The Bland and Altman plots for mean daily intakes for six of the 35 food groups (“rice, pasta, grains, and starches”, “yoghurts”, “eggs and egg dishes”, “other vegetables”, “fish and fish products”, “red meat”) are presented in Figure 4. The mean difference between the methods (bias) was small for all six food groups. Overall, these plots indicate good agreement between the two methods for the assessment of “rice, pasta, grains, and starches”, “yoghurts”, “eggs and egg dishes”, and “red meat”. Poorer agreement was observed for “other vegetables” and “fish and fish products/dishes”, with more than 5% of cases (n=7) outside the limits of agreement for both food groups [43].

SCC and cross-classification of mean daily food group intakes are presented in Table 6. The mean SCC for food group intake between the two FFQs was .68 and SCCs ranged from .41 for “savories” (lasagna, pizza) to .90 for “other fruits” (apples, pears, citrus fruits). High correlations (SCCs≥.5) were observed for 32 of the 35 food groups. Overall, ranking participants into quartiles of “exact agreement” was lowest for “cakes, buns, and pastries” (35%) and highest for “bananas” (73%). The percentage of participants cross-classified into quartiles of “exact agreement plus adjacent” was lowest for “cakes, buns, and pastries” (77%) and highest for “other fruits” (apples, pears, citrus fruits) (99%)*.* The mean percentage misclassified for all food groups was 12%.

**Table 5:** Mean daily food group intakes estimated by printed EPIC-Norfolk FFQ^a^and online Food4Me FFQ (n=113).

Food group	EPIC (grams), mean (SD)	Food4Me (grams), mean (SD)	*P* value^b^
Rice, pasta, grains, and starches	87.26 (74.30)	99.03 (87.54)	.28
Savories (lasagna, pizza)	17.69 (17.40)	34.85 (29.75)	<.001
White bread (rolls, tortillas, crackers)	13.65 (19.69)	43.93 (69.54)	<.001
Wholemeal and brown breads and rolls	28.20 (37.76)	54.28 (67.59)	<.001
Breakfast cereals and porridge	50.51 (50.67)	93.91 (91.64)	<.001
Biscuits	6.43 (10.56)	18.94 (25.06)	<.001
Cakes, pastries, and buns	17.76 (24.47)	19.70 (18.55)	.50
Milk	280.97 (166.19)	240.53 (178.04)	.08
Cheeses	17.36 (22.68)	24.38 (29.29)	.045
Yoghurts	29.20 (38.112)	60.98 (99.82)	.002
Ice cream, creams, and desserts	16.60 (28.48)	12.61 (17.44)	.21
Eggs and egg dishes	18.63 (19.62)	34.92 (36.39)	<.001
Fats and oils (eg, butter, low-fat spreads, hard cooking fats)	10.34 (10.38)	14.75 (13.00)	.005
Potatoes and potato dishes	47.39 (35.00)	68.18 (63.48)	.003
Chipped, fried, and roasted potatoes	11.51 (15.43)	14.13 (17.30)	.23
Peas, beans, and lentils and vegetable and pulse dishes	25.46 (23.91)	30.13 (31.56)	.21
Green vegetables	26.31 (27.96)	28.06 (35.97)	.68
Carrots	18.93 (20.84)	23.83 (23.43)	.10
Salad vegetables (eg, lettuce)	13.00 (14.45)	10.28 (10.97)	.11
Other vegetables (eg, onions)	96.05 (53.06)	111.62 (96.73)	.14
Tinned fruit or vegetables	15.30 (19.10)	18.84 (26.22)	.25
Bananas	52.00 (55.01)	61.54 (70.20)	.26
Other fruits (eg, apples, pears, oranges)	156.69 (140.10)	218.90 (206.63)	.01
Nuts and seeds, herbs and spices	3.54 (6.67)	2.48 (5.79)	.19
Fish and fish products/dishes	29.99 (31.68)	48.29 (48.70)	.001
Bacon and ham	12.76 (16.81)	12.11 (14.33)	.75
Red meat (eg, beef, veal, lamb, pork)	26.87 (31.75)	41.31 (39.25)	.003
Poultry (chicken and turkey)	31.47 (37.20)	42.79 (53.92)	.07
Meat products (eg, burgers, sausages, pies, processed meats)	9.15 (10.18)	19.76 (23.18)	<.001
Alcoholic beverages	104.80 (182.33)	200.32 (261.08)	.002
Sugars, syrups, preserves, and sweeteners	8.15 (10.21)	7.04 (9.53)	.40
Confectionary and savory snacks	23.87 (32.88)	25.31 (24.53)	.71
Soups, sauces, and miscellaneous foods	51.83 (52.32)	83.76 (84.80)	.001
Teas and coffees	533.78 (398.43)	472.08 (403.98)	.25
Other beverages (eg, fruit juices, carbonated beverages, squash)	109.73 (123.90)	213.66 (204.12)	<.001

^a^FFQ: food frequency questionnaire.

^b^All *P* values were derived using independent samples *t* tests.

**Table 6:** Spearman’s correlation coefficients (SCC) and cross-classification of quartiles of food group intake.

Food group	SCC	Exact agreement^a^(%)	Exact agreement + adjacent^b^(%)	Disagreement^c^(%)
Rice, pasta, grains, and starches	.76	55	94	6
Savories (lasagna, pizza)	.41	36	79	21
White bread (rolls, tortillas, crackers)	.55	42	83	17
Wholemeal and brown breads and rolls	.74	54	92	8
Breakfast cereals and porridge	.76	62	93	7
Biscuits	.67	45	90	13
Cakes, pastries, and buns	.48	35	77	23
Milk	.55	49	80	20
Cheeses	.66	50	85	15
Yoghurts	.70	58	89	11
Ice cream, creams, and desserts	.49	43	81	19
Eggs and egg dishes	.59	43	83	17
Fats and oils (eg, butter, low-fat spreads, hard cooking fats)	.64	43	86	14
Potatoes and potato dishes	.61	44	83	17
Chipped, fried, and roasted potatoes	.63	45	88	12
Peas, beans, and lentils and vegetable and pulse dishes	.66	49	83	17
Green vegetables	.76	56	91	9
Carrots	.65	51	85	15
Salad vegetables (eg, lettuce)	.58	50	84	16
Other vegetables (eg, onions)	.67	47	90	10
Tinned fruit or vegetables	.72	53	90	10
Bananas	.88	73	96	4
Other fruits (eg, apples, pears, oranges)	.90	70	99	1
Nuts and seeds, herbs and spices	.68	56	83	17
Fish and fish products/dishes	.73	51	90	10
Bacon and ham	.75	50	91	9
Red meat (eg, beef, veal, lamb, pork)	.69	50	93	7
Poultry (chicken and turkey)	.69	48	83	17
Meat products (eg, burgers, sausages, pies, processed meats)	.67	53	87	13
Alcoholic beverages	.87	66	95	5
Sugars, syrups, preserves, and sweeteners	.79	62	92	8
Confectionary and savory snacks	.66	53	88	12
Soups, sauces, and miscellaneous foods	.53	43	82	18
Teas and coffees	.77	59	93	7
Other beverages (eg, fruit juices, carbonated beverages, squash)	.79	60	92	8

^a^Exact agreement: % of cases cross-classified into the same quartile.

^b^Exact + adjacent agreement: % of cases cross-classified into the same or adjacent quartile.

^c^Disagreement: % of cases cross-classified 2 quartiles apart.

## Discussion

### Principal Results and Comparisons With Other Work

The present study demonstrates the development of a novel online FFQ and its comparison with the validated printed EPIC-Norfolk FFQ [35]. The Food4Me FFQ was developed to capture dietary intake in an online fashion. It was designed to include a range of portion size options and incorporated food photographs to aid food recognition and portion size estimation.

Overall, the current results demonstrate good agreement between the online Food4Me FFQ and the printed EPIC-Norfolk FFQ for both nutrient and food group intakes. Cross-classification of daily energy and nutrient intakes showed good agreement between the two FFQs indicating that the online Food4Me FFQ generates ranks of dietary intakes that are highly comparable with the previously validated printed EPIC-Norfolk FFQ. Similar to many previous published studies, classification into exact plus adjacent quartiles ranged from 77% to 97% and exact disagreement/misclassification ranged between 0% and 5% [21,28,44,45]. Furthermore, Bland and Altman plots demonstrated an acceptable level of agreement between the two methods for energy and total fat and carbohydrate intakes as a percentage of total energy.

In the present study, there were moderate to high correlation coefficients, ranging from .43 to .86, between the methods for individual nutrients. For the majority of nutrients analyzed, the correlation coefficients were within the ranges recommended by Willet et al [46] and Masson et al [47]; 25 out of 29 of the nutrients had a correlation ≥.50 of which four had a correlation >.70, indicating that estimates of intake obtained using the Food4Me FFQ are strongly correlated with those from the printed EPIC-Norfolk FFQ. The mean correlation coefficient was .60, which is within the range of those reported in the literature [16,32]. Furthermore, the ranges of correlation coefficients obtained in the present study are similar to those reported by Kristiansen et al [48]. The latter authors compared estimates of nutrient intake obtained at two time points by a semi-quantitative FFQ that had undergone slight modifications and obtained correlations ranging from .43 to .75.

Analyses at the food group level also showed good agreement between the EPIC-Norfolk and Food4Me FFQs. Validation studies of FFQs examining food group intakes have reported SCC ranging from .46 to .87 [49] and .14 to .90 [50]. In the current study, correlation coefficients >.50 were obtained for the majority of food groups showing that the Food4Me FFQ has reasonable ranking ability for food group intake estimates, and is comparable with the EPIC-Norfolk FFQ. Good agreement between the two methods for quartile classification of food group intakes was also observed with more than 75% of participants correctly classified into the same or adjacent quartiles for each of the food groups analyzed.

While we have demonstrated that the online Food4Me FFQ shows good agreement with the printed EPIC-Norfolk FFQ overall, some disagreement was observed between the two FFQs, particularly in relation to energy intakes. Similar to findings by Beasley et al [32], in the current study, the online FFQ yielded higher estimates for mean nutrient intakes in comparison to the printed FFQ. However, the percentage of participants classified as under-reporters was 33.6% higher with the printed EPIC-Norfolk FFQ compared with the online Food4Me FFQ, indicating that the Food4Me FFQ may have a greater ability to capture usual dietary intake than the EPIC-Norfolk FFQ.

Multiple factors could have contributed to the discrepancies observed in mean daily intakes and the occurrence of under-reporting between the two methods. First, numerous differences in food group consumption were observed between the two FFQs. For 17 of the 35 food groups (49%), mean daily intakes were significantly higher with the online Food4Me FFQ compared with the printed EPIC-Norfolk FFQ. In addition, Bland and Altman plots demonstrated a poor level of agreement between the methods for 20 of the 35 food groups (57%). The low level of agreement for food group consumption between the two methods is most likely attributable to differences in reported food intake between both FFQs. The Food4Me FFQ included an additional 27 food items not present in the printed EPIC-Norfolk FFQ and as a result several of the 35 food groups analyzed in the current study did not contain equal numbers of food items for both methods. For example, the food group “fish and fish products/dishes” consisted of 10 food items for the Food4Me FFQ compared with six food items from the EPIC-Norfolk FFQ. Such variances in the number of food items aggregated into food groups (for both FFQs) could partly explain the differences observed in mean daily food group intakes between the two methods.

The additional 27 food items listed in the Food4Me FFQ would have offered participants a greater selection of food items and, as a result, both food group consumption and subsequent nutrient intakes may be more reflective of true dietary intake. However, it is also possible that the additional food items included in the Food4Me FFQ may have resulted in an overestimation of consumption frequency for particular nutrients and food groups. This can occur when several food items of a single food group are listed in a questionnaire [50]. Furthermore, despite both FFQs being conducted within 4 weeks (to minimize temporal changes in dietary intakes), there is also the potential that the consumption of a particular food item/group was recalled/reported with one FFQ and not the other (impacting on nutrient and energy intakes).

A second factor potentially contributing to the differences observed between the two FFQs is portion size estimation. The online Food4Me FFQ incorporates a selection of portion sizes for the majority of food items, as opposed to applying a standard one to each food item. Presuming all participants consume standard portion sizes is a generalization and the use of standard portion sizes in heterogeneous population groups is likely to result in additional inaccuracies [51]. Printed questionnaires are limited in their ability to collect complex information (eg, portion size consumption) due to practical restrictions in-built in the questionnaire format [52]. It is more feasible to obtain complex information with online questionnaires, which can be designed to embed multiple photographs to aid with portion size estimation. However, questionnaires with multiple portion size options will exhibit more variability compared to those without variable portion sizes (which may be a truer estimate of actual intakes) [50]. When examining food group intakes, Ocke et al [53] found that the use of photographs for dairy desserts resulted in an overestimation of milk and milk products. Other studies have reported that photographs have a positive effect on the respondents’ ability to accurately estimate portion sizes [14,32] and further investigation is needed to determine whether the use of photographs in the Food4Me FFQ aids portion size estimation and food recognition.

Third, the differences we observed between the two methods in relation to daily nutrient intakes may have been related to variances in the nutritional composition databases utilized in both methods, as the nutritional composition database used to calculate daily nutrient intakes is more up to date than that used for analysis of the printed EPIC-Norfolk FFQ [41]. The nutritional composition database used in the EPIC-Norfolk FFQ is based on the revised and extended 5^th^edition of McCance and Widdowson’s *The Composition of Foods* plus supplemental volumes, while the nutritional composition data for the Food4Me FFQ is based on the 6^th^and 5^th^editions of McCance and Widdowson’s *The Composition of Foods* plus all nine supplemental volumes [38,41].

### Strengths and Limitations

The strengths of the current study include the cross-over design and adequate sample size [46,54]. In addition, all participants included in the analyses completed both questionnaires within 1 month, minimizing the likelihood of changes in dietary intake. Limitations of the current study include the use of self-reported weight and height measurements for all participants. BMR was estimated using the Henry equations and therefore any errors in these self-reported measurements could have impacted on the frequency of under-reporting that we observed in the results. Another limitation of the current study is that the majority of participants involved were recruited from within the universities and are therefore representative of a convenient sample rather than a nationally representative sample. As the accuracy of any dietary assessment method using self-report depends ultimately on the cooperation and motivation of the participants, further testing of the Food4Me FFQ will be necessary to establish its wider utility.

### Conclusions

In conclusion, the online Food4Me FFQ has good agreement with the previously validated printed EPIC-Norfolk FFQ for assessing both nutrient and food group intakes of healthy young adults. While some differences were observed between the methods, particularly in relation to mean daily nutrient and food group intakes, good agreement was observed at both the nutrient and food group level using a variety of analyses. The most common use of an FFQ is not to measure absolute intake but to rank individuals by their food and nutrient intakes [21]. In the current study, the Food4Me FFQ was able to generate ranks of nutrient and food group intakes that were highly comparable with the validated EPIC-Norfolk FFQ, with levels of agreement from quartile cross-classification similar to many previous published studies. Therefore, the good agreement with the printed EPIC-Norfolk FFQ combined with its ease of use make the online Food4Me FFQ a useful tool for ranking individuals based on their nutrient intake and could be potentially valuable for use in other epidemiological studies.

## Multimedia Appendix 1

Food4Me Food Frequency Questionnaire screenshots.

## Abbreviations

BMI: body mass index
BMR: basal metabolic rate
EER: estimated energy requirements
EPIC: European Prospective Investigation of Cancer
FFQ: food frequency questionnaire
MUFA: monounsaturated fatty acids
NANS: National Adult Nutrition Survey
PUFA: polyunsaturated fatty acids
SCC: Spearman’s correlation coefficient
SFA: saturated fatty acids
TE: total energy
Vitamin A (RE): vitamin A retinol equivalents
